# Risk Factors and Predictors for Functional Outcome and Complication Rate in Total Hip Arthroplasty through Minimally Invasive and Conventional Approaches: A Systematic Review and Meta-Regression Analysis of 41 Randomized Controlled Trials

**DOI:** 10.3390/jcm12185895

**Published:** 2023-09-11

**Authors:** Nikolai Ramadanov, Marko Ostojic, Philip Lazaru, Kuiliang Liu, Robert Hable, Polina Marinova-Kichikova, Dobromir Dimitrov, Roland Becker

**Affiliations:** 1 Center of Orthopaedics and Traumatology, Brandenburg Medical School, University Hospital Brandenburg an der Havel, 14770 Brandenburg an der Havel, Germany; roland.becker@mhb-fontane.de; 2 Department of Orthopedics, University Hospital Mostar, 88000 Mostar, Bosnia and Herzegovina; dr.ostojic@gmail.com; 3 Department of General and Visceral Surgery, Minimally Invasive Surgery and Coloproctology, St. Marien Hospital, 12249 Berlin, Germany; philip.lazaru@gmail.com; 4 Department for Orthopaedics and Trauma Surgery, Siloah St. Trudpert Hospital, 75179 Pforzheim, Germany; kuiliang.liu@siloah.de; 5 Faculty of Applied Computer Science, Deggendorf Institute of Technology, 94469 Deggendorf, Germany; robert.hable@th-deg.de; 6 Department of Surgical Propaedeutics, Faculty of Medicine, Medical University of Pleven, 5800 Pleven, Bulgaria; polina_g.marinova@abv.bg; 7 Department of Surgical Diseases, Faculty of Medicine, Medical University of Pleven, 5800 Pleven, Bulgaria; dobri_dimitrov@abv.bg

**Keywords:** total hip arthroplasty, meta-regression, minimally invasive, conventional approach

## Abstract

Objective: To investigate and identify risk factors and predictors for the difference in functional outcome and complications between total hip arthroplasty (THA) through minimally invasive and conventional approaches, using a meta-regression analysis of randomized controlled trials (RCTs). Methods: A systematic review of the literature up to 31 July 2022 was performed. A meta-regression was conducted based on a random effects meta-analysis using the Hartung–Knapp–Sidik–Jonkman method. Results: A total of 41 RCTs with 3607 patients were found. The following predictors of HHS ≥ 6 months postoperatively were identified: patient age (predictor estimate = 0.14; *p* < 0.01), avascular necrosis of the femoral head (predictor estimate = −0.03; *p* = 0.04); incision length (predictor estimate = −0.82; *p* < 0.01). The following predictors of complication rate were identified: osteoarthritis (predictor estimate = 0.02; *p* = 0.02); femoral neck fracture (predictor estimate = −0.02; *p* = 0.02); SuperPATH (predictor estimate = −1.72; *p* < 0.01). Conclusions: Patient age, avascular necrosis of the femoral head, and incision length were identified as predictors of the effect size of the HHS ≥ 6 months postoperatively; and osteoarthritis, femoral neck fracture, and SuperPATH as predictors of the effect size of the complication rate. Based on these findings, we recommend that more frequent use of minimally invasive THA in elderly patients should be considered. Level of evidence I: a systematic review of all relevant randomized controlled trials. Registered in PROSPERO on 10 August 2022 (CRD42022350287).

## 1. Introduction

Total hip arthroplasty (THA) is one of the most effective and successful procedures in orthopaedic surgery [1]. THA relieves pain, restores function to the hip joint, and improves the patient’s overall quality of life. There are several indications for THA: symptomatic hip osteoarthritis, avascular necrosis of the femoral head (ANFH), hip dysplasia, and inflammatory arthritic conditions. As the world’s population ages, the number of hip joint disorders is increasing [2]. Approximately 240 million people worldwide have symptomatic osteoarthritis [3,4]. Almost 10% of patients over the age of 45 have radiographic evidence of symptomatic hip osteoarthritis [5]. The prevalence of ANFH is two per 100,000 people [6]. It is more common in males, with the highest prevalence in men aged 25 to 44 years and women aged 55 to 75 years [7]. The absolute number of femoral neck fractures is expected to increase to 2.6 million in 2025 and 4.5 million in 2050 [8]. According to guidelines, the operating surgeon can choose between endoprosthetic and femoral head-preserving procedures for surgical treatment [9]. Femoral neck fractures in elderly patients are increasingly treated with THA.

Surgical approaches to the hip joint are divided into six types based on the anatomical relationship to the greater trochanter: anterior, anterolateral, lateral (transgluteal or transtrochanteric), posterior, posterolateral, and superior. Minimally invasive or muscle-sparing surgical approaches to the hip joint are modifications of these conventional approaches that must satisfy two conditions: the preservation of musculotendinous structures, and a short incision length (≤10 cm). The direct anterior approach (Smith-Petersen), the anterolateral approach (modified Watson–Jones), the direct superior approach with SuperPATH technique and the two-incision approach are described as minimally invasive [10,11,12,13]. The advantages of minimally invasive approaches include less pain, lower blood loss, and faster recovery due to less surgical tissue trauma [14]. Limited visibility during exposure has been highlighted as a disadvantage of minimally invasive approaches. Sometimes, this disadvantage has to be compensated for by excessive intraoperative wound retraction or by an unphysiological positioning of the leg, which in some cases can lead to complications [15,16].

Unfortunately, not all patients benefit to the same extent after THA. The reasons for this are not yet clear. There are several studies [17,18,19,20,21,22,23,24] and systematic reviews [25,26,27,28,29] that have shed light on some risk factors and predictors of THA outcome, but there is still no meta-regression analysis on this topic in the specialist literature. In particular, there is no single study that examines the risk factors and predictors of differences in outcome between minimally invasive and conventional approach THA.

We aimed to investigate and identify risk factors and predictors for the effect size of functional outcome and complications after THA through minimally invasive and conventional approaches by performing a meta-regression analysis of randomized controlled trials (RCTs).

## 2. Materials and Methods

### 2.1. Search Strategy and Inclusion Criteria

**Figure 1:**
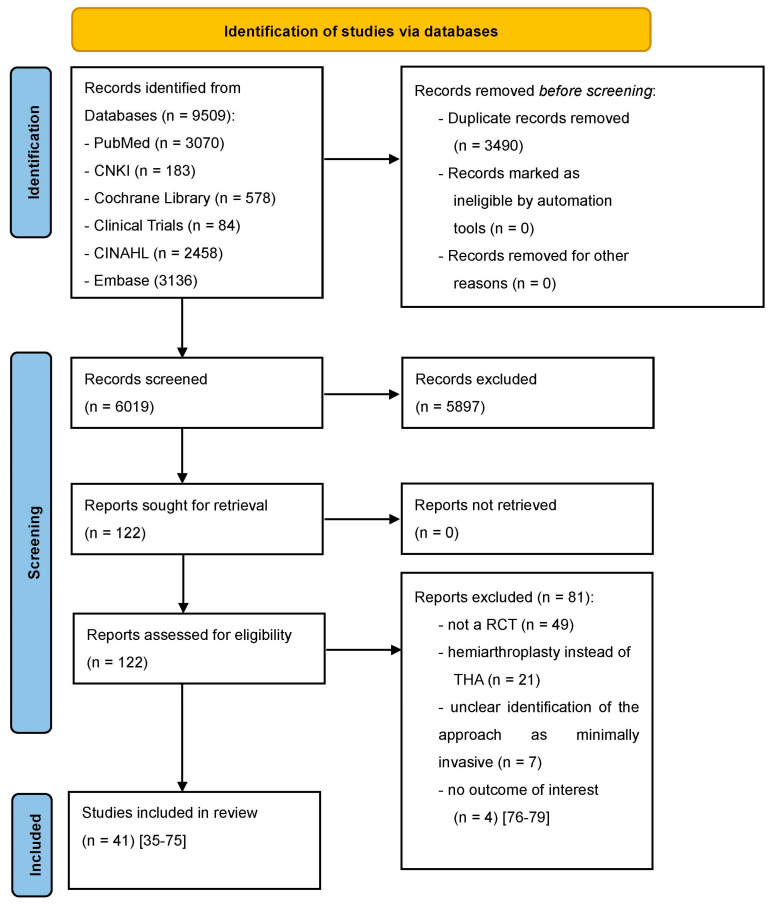
PRISMA flow diagram of the search results and selection according to our inclusion criteria. CNKI: China National Knowledge Infrastructure; CINAHL: Cumulative Index to Nursing and Allied Health Literature; RCT: randomized controlled trial; THA: total hip arthroplasty [35,36,37,38,39,40,41,42,43,44,45,46,47,48,49,50,51,52,53,54,55,56,57,58,59,60,61,62,63,64,65,66,67,68,69,70,71,72,73,74,75,76,77,78,79].

Our study protocol was registered in PROSPERO on 10 August 2022 (CRD42022350287). Two independent reviewers (NR,PL) searched the following databases for relevant manuscripts up to 31 July 2022: PubMed, China National Knowledge Infrastructure (CNKI), The Cochrane Library, Clinical trials, Cumulative Index to Nursing and Allied Health Literature (CINAHL), and Embase. We used the MeSH terms ‘minimally invasive’, ‘muscle-sparing’, ‘SuperPATH’, ‘direct anterior’, ‘two incision’, ‘conventional approaches’, ‘THA’, ‘THR’, ‘hip arthroplasty’, and ‘hip replacement’ with a BOOLEAN search strategy and adapted them to the syntax of the databases. There were no restrictions on publication language. We did not include old RCTs, published before 2010. The reason for this is that there have been many improvements in THA over the last decades. Older THA methods have lost their importance today, making it difficult to compare them with newer THA methods in a meta-regression analysis. A Chinese-speaking reviewer (KL) helped by translating Chinese articles. In addition to this search strategy, we performed a manual review of the reference lists of relevant systematic reviews. We did not review grey literature. Inclusion criteria were: (i) randomized controlled trials (RCT) with (ii) human participants (without demographic restrictions such as patient age, sex, body mass index (BMI), etc.) with hip disease (osteoarthritis, dysplasia, ANFH) or femoral neck fracture, who were treated with (iii) THA through minimally invasive approaches or conventional approaches. Exclusion criteria were: no outcome of interest, hip replacement with hemiarthroplasty, unclear identification of the approach as minimally invasive. Hip approaches for THA were accepted as minimally invasive approaches under at least one of the following two conditions: (1) If the approaches were minimally invasive by definition; this means that the approach per se is known to be muscle and tendon sparing and has an incision length ≤ 10 cm. (2) In other cases, we referred to the authors’ assessment if an approach was explicitly described as minimally invasive in their RCT. The literature search is presented in a PRISMA flowchart diagram (Figure 1).

### 2.2. Data Extraction

Two independent reviewers extracted the following information from each RCT, according to the PRISMA guidelines: RCT details (e.g., study design, treatment protocol, duration, number of patients, number of hips operated, year of publication, and risk of bias), primary patient characteristics (e.g., age, sex, BMI, preoperative Harris Hip Score (HHS), and indication for surgery), intervention (e.g., approach, use of bone cement, table position, use of traction table, operation time, incision length, intraoperative blood loss, cup inclination angle, laboratory parameters), patient outcome (e.g., postoperative HHS and complication rate). In the case of missing data, a letter requesting additional information was sent to the corresponding authors. If information on standard deviation was missing, it was calculated by imputation [30].

### 2.3. Outcome Parameters

We focused on the two main THA outcome parameters: the functional outcome parameter ‘HHS’ and the ‘complication rate’. The HHS was developed to assess the outcome of hip surgery [31]. This score accumulates points from the assessment of four aspects: pain, function, degree of deformity, and range of motion of the hip. The higher the total score, the better the outcome, with a range of total scores from 0 to 100. The second outcome parameter was the complication rate. We considered the following relevant types of complications: dislocation, infection, intraoperative periprosthetic fracture, deep vein thrombosis of the lower extremity, and haematoma. To obtain more consistent data, we summarized the reported data into HHS ≤ 3 months postoperatively and HHS ≥ 6 months postoperatively. If the RCT reported more than one value for one outcome parameter, we used the most recent record of short-term HHS and short-term complications.

### 2.4. RCT Quality Assessment

Risk of bias and level of evidence were assessed according to the Cochrane’s risk of bias 2 (RoB 2) tool [32] and the recommendations of the GRADE system [33]. We assessed the RCTs for publication bias, by using the Egger’s regression intercept test for asymmetry of the funnel plots. Statistical significance was set at a *p*-value < 0.05. We presented the results in funnel plots to find evidence of publication bias. In the funnel plot, the horizontal axis (‘*x*-axis’) shows the estimated effect size of the RCTs and the ‘*y*-axis’ shows the estimated standard error of the RCTs (=measure of the uncertainty of the estimated effect size). The dashed vertical line is the overall effect estimated from the meta-analysis of all RCTs. Ideally, the RCTs should be symmetrically distributed within the triangle.

### 2.5. Data Synthesis and Statistical Analysis

We performed a meta-regression based on a random effects meta-analysis using the Hartung–Knapp–Sidik–Jonkman method for both continuous and nominal study level covariates [34]. We fitted regression models with single covariates and assessed heterogeneity using Cochrane’s $Q_{E}$ test (*p* value < 0.10 indicates heterogeneity). The effect of covariates was assessed using the $Q_{M}$ Wald-type test on model coefficients.

The forest plots show the measures of treatment effect between minimally invasive THA and conventional approach THA, labelled ‘experimental group’ and ‘control group’ respectively. Mean differences (MDs) with 95% confidence intervals (CIs) were calculated for the continuous outcome parameter HHS and odds ratios (ORs) with 95% CIs were calculated for the dichotomous outcome parameter complication rate. A positive MD and an OR of less than 1 favoured the experimental group. In addition, the results of the common effect model are also shown in the forest plots.

The bubble plots illustrate the meta-regression results. The value of the predictor for each RCT is plotted on the horizontal axis (‘*x*-axis’) and the effect size is plotted on the vertical axis (‘*y*-axis’). Each bubble in the bubble plots corresponds to one RCT. The size of the bubbles indicates the weight with which the study contributes to the overall result. The black solid line is the regression line. The slope of the regression line corresponds exactly to the effect size of the predictor on the outcome variable. If the predictor has a strong influence on the outcome variable, then the slope of the regression line is large (steep line). If the predictor has no influence on the outcome variable, the regression line is flat and more or less parallel to the horizontal zero line.

A professional statistician (RH) performed all statistic calculations using the R packages meta and metaphor, with minor assistance from the first author (NR). We reported THAs rather than patients because in some RCTs patients received bilateral THAs.

## 3. Results

According to our inclusion criteria, we found a total of 41 RCTs [35,36,37,38,39,40,41,42,43,44,45,46,47,48,49,50,51,52,53,54,55,56,57,58,59,60,61,62,63,64,65,66,67,68,69,70,71,72,73,74,75] in the systematic review of the literature (Figure 1). The RCTs included 3630 THAs in 3607 patients. Of these 3607 patients, 1734 (48.07%) underwent minimally invasive THA surgery and 1873 (51.93%) underwent conventional THA surgery. Of these 1734 patients in the minimally invasive group, 765 (44.12%) patients from 16 RCTs were operated through a direct anterior approach [35,36,37,38,39,40,53,54,55,57,60,62,63,65,66,75], 83 (4.79%) patients from 1 RCT were operated through a MicroHip approach [41], 143 (8.25%) patients from 3 RCTs were operated through a minimally invasive posterior approach [42,46,69], 140 (8.07%) patients from 4 RCTs were operated through a minimally invasive anterolateral approach [51,56,64,72], 25 (1.44%) patients from 1 RCT were operated through a minimally invasive lateral approach [67], and 578 (33.33%) patients from 16 RCTs were operated through SuperPATH [43,44,45,47,48,49,50,52,58,59,61,68,70,71,73,74]. The mean age of the patients was 64.93 years (range: 51.00-89.10), and 1766 patients (48.96%) were male. The mean BMI of the patients was 26.34 kg/m^2^ (range: 21.50–34.60). The mean preoperative HHS was 46.30 points (range: 15.40–84.00). The most common surgical indications were osteoarthritis with 2287 (65.56%), femoral neck fracture with 665 (19.07%), ANFH with 505 (14.48%) and dysplasia with 30 (0.86%) of a total of 3487 diagnoses. Only 24 [35,36,37,39,40,41,42,46,51,53,54,55,56,57,60,62,63,64,66,67,69,70,72,73] of the 41 RCTs provided information on the use of bone cement. Of these 24 RCTs, 10 RCTs [36,40,41,42,46,51,53,54,60,63] used cemented prosthesis anchorage. These 10 RCTs included 1125 THAs, of which 687 THAs (61.07%) were operated with the use of bone cement. No bone cement was used in the remaining 14 RCTs. The mean operation time was 77.91 min. (range: 36.00–125.30). The mean incision length was 10.98 cm (range: 5.80–19.30). The mean incision length of conventional approach THA was 12.86 cm, and the mean incision length of minimally invasive THA was 9.10 cm. The mean intraoperative blood loss was 340.09 mL (range: 71.90–1644.00). The mean acetabular cup inclination angle was 42.60° (range: 37.00–50.10). The mean C-reactive protein (CRP) level 1–3 days postoperatively was 80.61 mg/L (range: 11.40–178.00). The mean creatine kinase (CK) level 1–3 days postoperatively was 594.34 mg/L (range: 203.20–1035.25). Further details are shown in Table 1 and Table 2. Two of the RCTs had identical patient cohorts, providing different outcome parameters with different follow-up times [53,54]. In addition, two RCTs [52,71] included patients with bilateral THA (see Table 1). Two RCTs [52,64] did not report data on BMI and patient age separately for minimally invasive THA and conventional approach THA, but summarized them for the whole patient cohort (see Table 1).

### 3.1. Quality Assessment

**Figure 2:**
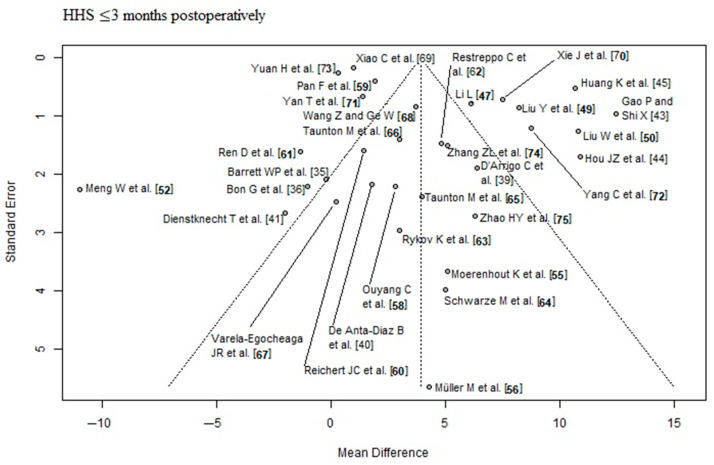
Funnel plot of the HHS ≤ 3 months postoperatively. Many RCTs [43,44,45,49,50,70,72] lie outside of the funnel plot triangle, especially in the upper right area, which detects a high risk of publication bias (Egger’s *p*-value = 0.03). HHS: Harris hip score [35,36,39,40,41,43,44,45,47,49,50,52,55,56,58,59,60,61,62,63,64,65,66,67,68,69,70,71,72,73,74,75].

**Figure 3:**
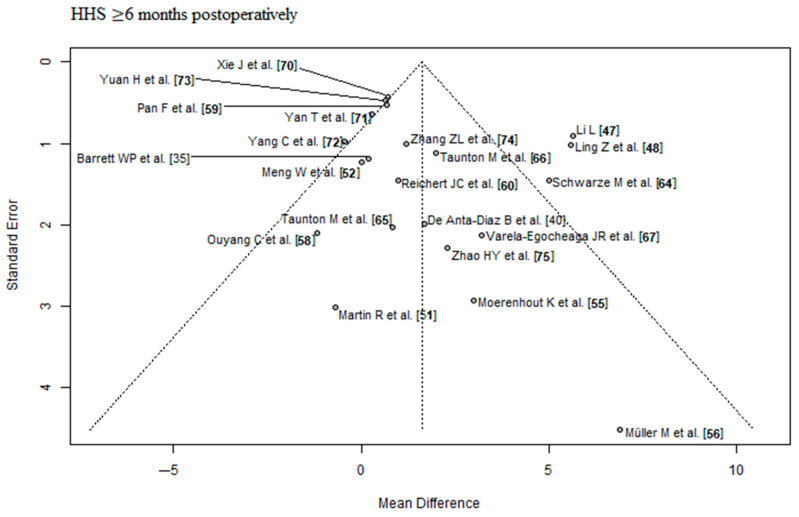
Funnel plot of the HHS ≥ 6 months postoperatively. Most of the RCTs lie inside of the funnel plot triangle, which detects a low risk of publication bias. HHS: Harris hip score [35,40,47,48,51,52,55,56,58,59,60,64,65,66,67,70,71,72,73,74,75].

**Figure 4:**
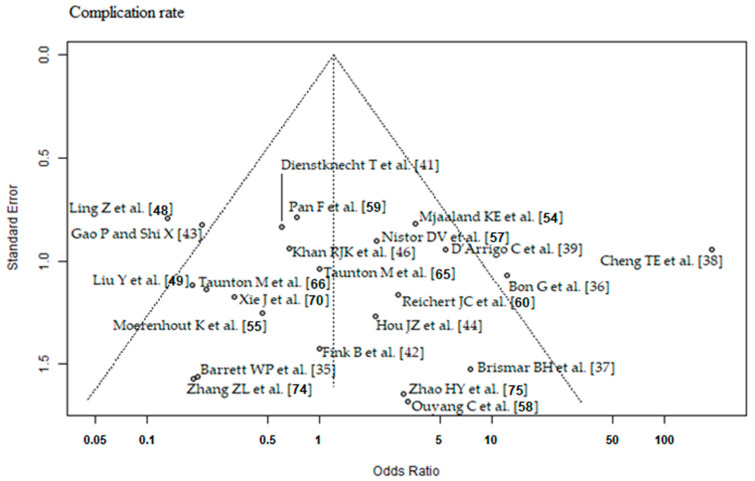
Funnel plot of the complication rate. Most of the RCTs lie inside of the funnel plot triangle, which detects a low risk of publication bias [35,36,37,38,39,41,42,43,44,46,48,49,54,55,57,58,59,60,65,66,70,74,75].

The outcome parameter HHS ≤ 3 months postoperatively showed a high risk of publication bias (Egger’s *p*-value = 0.03, Figure 2). In the corresponding funnel plot, there are many RCTs [43,44,45,49,50,70,72] outside of the triangle, especially in the upper right area. These RCTs show a relatively large positive effect with a relatively small standard error (i.e., with a relatively large apparent certainty). The outcome parameters HHS ≥ 6 months postoperatively and complication rate showed a low risk of publication bias (Egger’s *p*-value = 0.2, Figure 3; Egger’s *p*-value = 0.83, Figure 4, respectively). The risk of bias and the level of evidence assessment are shown in Table 3 and Table 4. According to the Cochrane’s Risk of Bias 2 (RoB 2) tool [32], 19 [35,37,38,40,41,43,45,47,48,49,57,59,60,61,63,64,67,68,73] out of 41 RCTs had a high risk of bias, 8 RCTs [44,51,56,65,66,69,71,74] had a moderate risk of bias, and 14 RCTs [36,39,42,46,50,52,53,54,55,58,62,70,72,75] had a low risk of bias. According to the recommendations of the GRADE system [33], the outcome parameters HHS ≤ 3 months postoperatively and complication rate showed low quality of evidence, and the outcome parameter HHS ≥ 6 months postoperatively showed moderate quality of evidence.

### 3.2. Meta-Analysis

#### 3.2.1. HHS ≤ 3 Months Postoperatively

**Figure 5:**
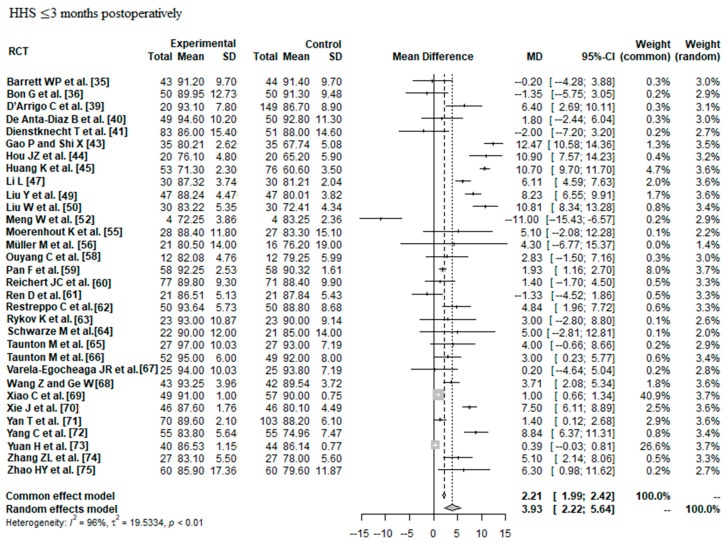
Forest plot of the HHS ≤ 3 months postoperatively. The MD of the summary measure has positive values, which favours minimally invasive THA (MD = 3.93, 95% CI 2.22 to 5.64). RCT: randomized controlled trial; SD: standard deviation; MD: mean difference; CI: confidence interval; HHS: Harris hip score [35,36,39,40,41,43,44,45,47,49,50,52,55,56,58,59,60,61,62,63,64,65,66,67,68,69,70,71,72,73,74,75].

Data on 2690 THAs were pooled from 32 RCTs (I^2^ = 96%, *p* < 0.01, Figure 5). The HHS ≤ 3 months postoperatively of minimally invasive THA was 3.93 points higher than the HHS ≤ 3 months postoperatively of conventional approach THA (MD = 3.93, 95% CI 2.22 to 5.64).

#### 3.2.2. HHS ≥ 6 Months Postoperatively

**Figure 6:**
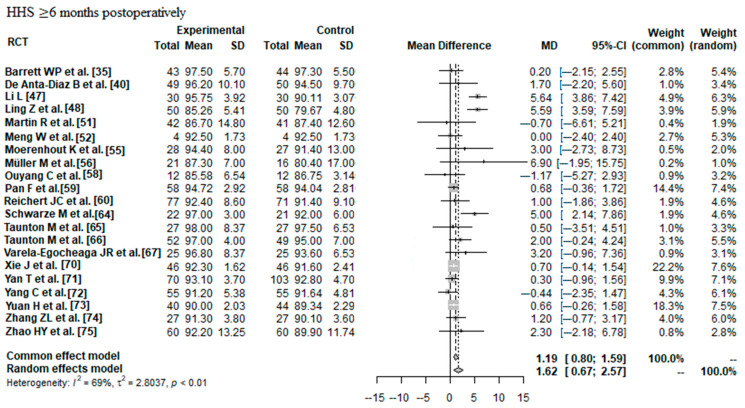
Forest plot of the HHS ≥ 6 months postoperatively. The MD of the summary measure has positive values, which favours minimally invasive THA (MD = 1.62, 95% CI 0.67 to 2.57). RCT: randomized controlled trial; SD: standard deviation; MD: mean difference; CI: confidence interval; HHS: Harris hip score [35,40,47,48,51,52,55,56,58,59,60,64,65,66,67,70,71,72,73,74,75].

Data on 1698 THAs were pooled from 21 RCTs (I^2^ = 69%, *p* < 0.01, Figure 6). The HHS ≥ 6 months postoperatively of minimally invasive THA was 1.62 points higher than the HHS ≥ 6 months postoperatively of conventional approach THA (MD = 1.62, 95% CI 0.67 to 2.57).

#### 3.2.3. Complication Rate

**Figure 7:**
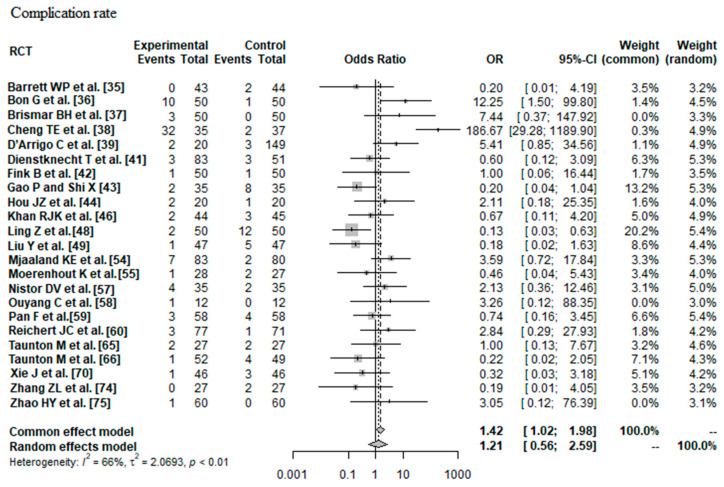
Forest plot of the complication rate. The 95% CI of the OR summary measure have a <1 and a >1 value, which means that there was no difference between minimally invasive and conventional approach THA (OR = 1.21, 95% CI 0.56 to 2.59). RCT: randomized controlled trial; SD: standard deviation; OR: odds ratio; CI: confidence interval; THA: total hip arthroplasty [35,36,37,38,39,41,42,43,44,46,48,49,54,55,57,58,59,60,65,66,70,74,75].

Data on 2152 THAs were pooled from 23 RCTs (I^2^ = 66%, *p* < 0.01, Figure 7). The complication risk of minimally invasive THA was indifferent compared with the complication risk of conventional approach THA (OR = 1.21, 95% CI 0.56 to 2.59).

### 3.3. Meta-Regression Analysis

#### 3.3.1. Risk Factors and Predictors of HHS ≤ 3 Months Postoperatively

The following predictors and risk factors were examined for their influence on HHS ≤ 3 months postoperatively: patient age, BMI, preoperative HHS, sex, osteoarthritis, femoral neck fracture, dysplasia, ANFH, surgical approach, operation time, incision length, intraoperative blood loss, acetabular cup inclination, CRP 1–3 days postoperatively, CK 1–3 days postoperatively, and use of bone cement. None of these predictors and risk factors were statistically significant (see Table 5).

#### 3.3.2. Risk Factors and Predictors of HHS ≥ 6 Months Postoperatively

**Figure 8:**
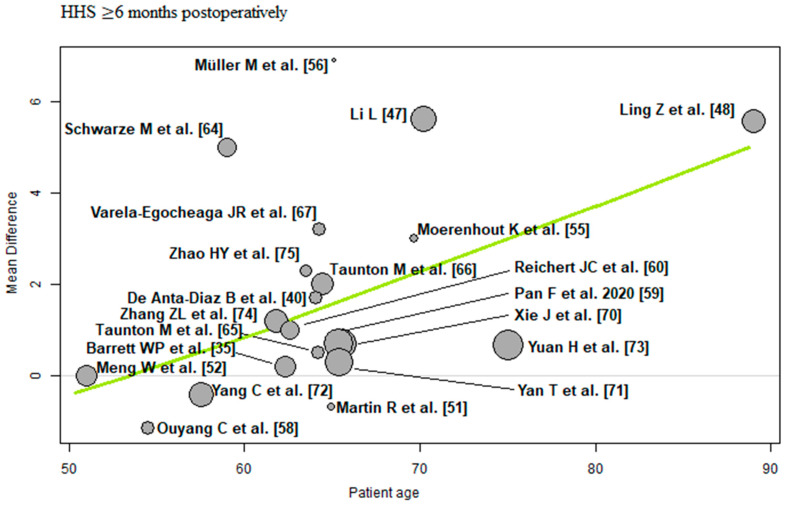
Bubble plot of the predictor patient age and the outcome parameter HHS ≤ 3 months postoperatively. The slope of the regression line (in green) is large (steep line), which shows that the predictor has a strong influence on the outcome variable. HHS: Harris hip score [35,40,47,48,51,52,55,56,58,59,60,64,65,66,67,70,71,72,73,74,75].

Patient age: Of the 21 RCTs [35,40,47,48,51,52,55,56,58,59,60,64,65,66,67,70,71,72,73,74,75] with 1698 THAs reporting patient age demographics, a positive association (predictor estimate = 0.14) was found between patient age and HHS ≥ 6 months postoperatively. For each 1-year increase in mean patient age, the effect size for HHS ≥ 6 months postoperatively increased by an average of 0.14 points (*p* < 0.01; Figure 8).

**Figure 9:**
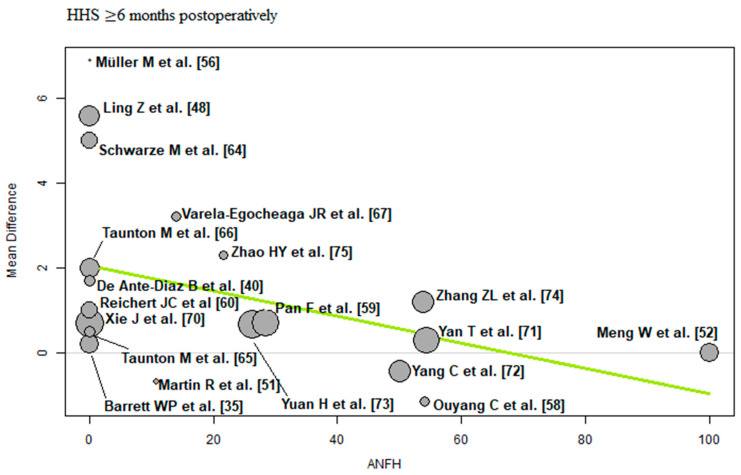
Bubble plot of the predictor ANFH and the outcome parameter HHS ≤ 3 months postoperatively. The slope of the regression line (in green) is large (steep line), which shows that the predictor has a strong influence on the outcome variable. HHS: Harris hip score; ANFH: avascular necrosis of the femoral head [35,40,48,51,52,56,58,59,60,64,65,66,67,70,71,72,73,74,75].

ANFH: Of the 19 RCTs [35,40,48,51,52,56,58,59,60,64,65,66,67,70,71,72,73,74,75] with 1594 THAs reporting ANFH, a negative association (predictor estimate = −0.03) was found between ANFH and HHS ≥ 6 months postoperatively. For every 1 percentage point increase in the incidence of ANFH, the effect size for HHS ≥ 6 months postoperatively decreased by an average of 0.03 points (*p* = 0.04; Figure 9).

**Figure 10:**
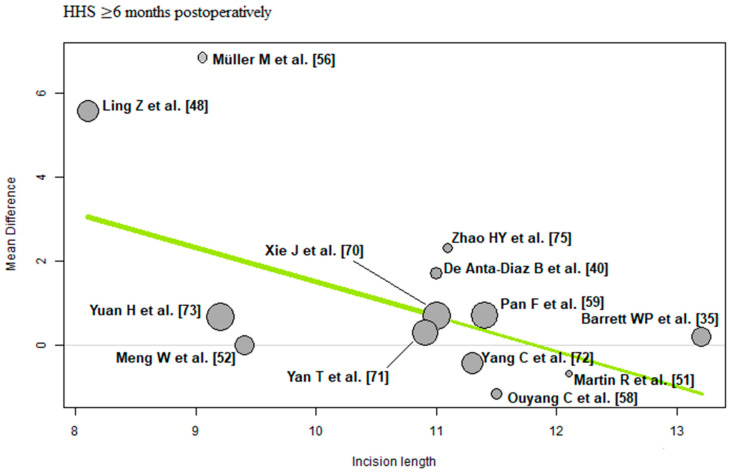
Bubble plot of the predictor incision length and the outcome parameter HHS ≤ 3 months postoperatively. The slope of the regression line (in green) is large (steep line), which shows that the predictor has a strong influence on the outcome variable. HHS: Harris hip score [35,40,48,51,52,56,58,59,70,71,72,73,75].

Incision length: Of the 13 RCTs [35,40,48,51,52,56,58,59,70,71,72,73,75] with 1133 THAs reporting incision length, there was a negative association (predictor estimate = −0.82) between incision length and HHS ≥ 6 months postoperatively. For each 1 cm increase in mean incision length, the effect size for HHS ≥ 6 months postoperatively decreased by an average of 0.82 points (*p* < 0.01; Figure 10).

The remaining risk factors and predictors (BMI, preoperative HHS, sex, osteoarthritis, femoral neck fracture, dysplasia, surgical approach, operation time, intraoperative blood loss, acetabular cup inclination, CRP 1–3 days postoperatively, CK 1–3 days postoperatively, and use of bone cement) were not statistically significant (see Table 5).

#### 3.3.3. Risk Factors and Predictors of Complication Rate

**Figure 11:**
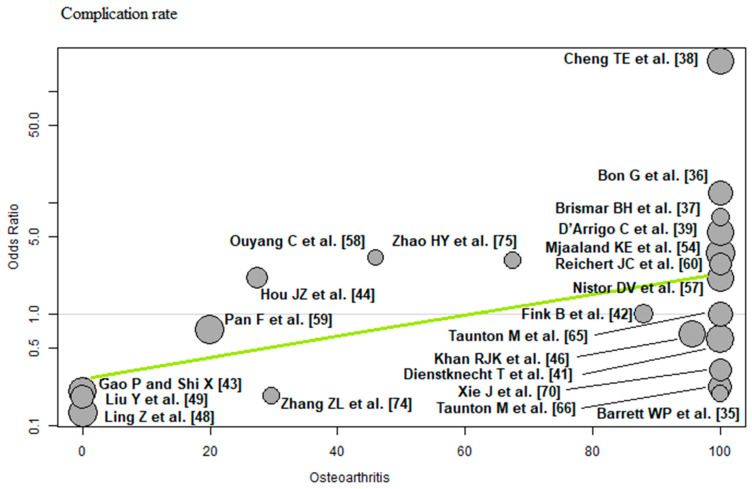
Bubble plot of the predictor osteoarthritis and the outcome parameter complication rate. The slope of the regression line (in green) is large (steep line), which shows that the predictor has a strong influence on the outcome variable [35,36,37,38,39,41,42,43,44,46,48,49,54,57,58,59,60,65,66,70,74,75].

Osteoarthritis: Of the 22 RCTs [35,36,37,38,39,41,42,43,44,46,48,49,54,57,58,59,60,65,66,70,74,75] with 2097 THAs reporting osteoarthritis, a positive association (predictor estimate = 0.02) was found between osteoarthritis and the complication rate. For each 1 percentage point increase in the incidence of osteoarthritis, the effect size for the complication rate increased by an average of 0.02 (*p* = 0.02; Figure 11).

**Figure 12:**
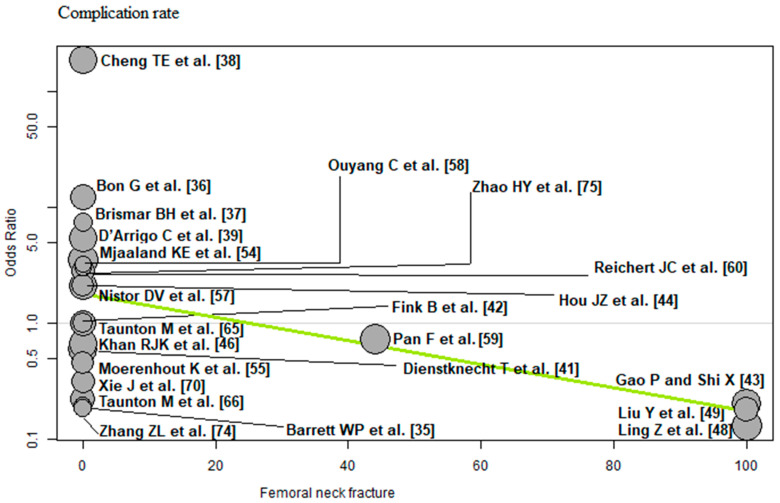
Bubble plot of the predictor femoral neck fracture and the outcome parameter complication rate. The slope of the regression line (in green) is large (steep line), which shows that the predictor has a strong influence on the outcome variable [35,36,37,38,39,41,42,43,44,46,48,49,54,55,57,58,59,60,65,66,70,74,75].

Femoral neck fracture: Of the 23 RCTs [35,36,37,38,39,41,42,43,44,46,48,49,54,55,57,58,59,60,65,66,70,74,75] with 2152 THAs reporting femoral neck fracture, a negative association (predictor estimate = −0.02) was found between femoral neck fracture and the complication rate. For each 1 percentage point increase in the incidence of femoral neck fracture, the effect size for the complication rate decreased by an average of 0.02 (*p* = 0.02; Figure 12).

**Figure 13:**
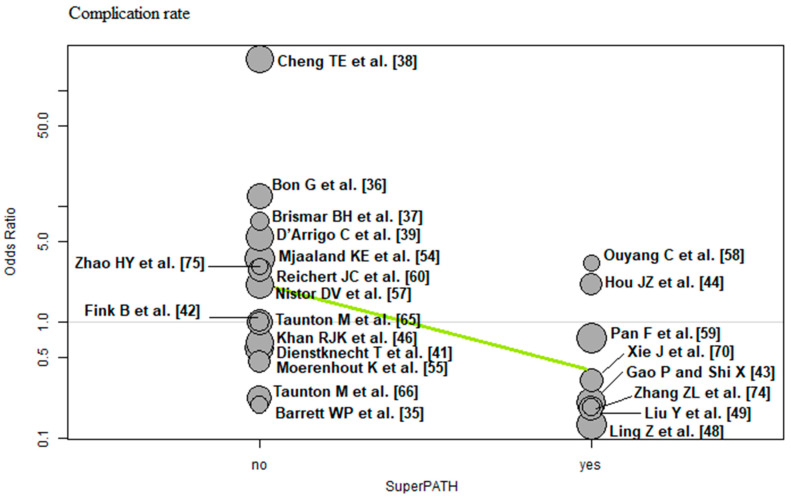
Bubble plot of the predictor SuperPATH and the outcome parameter complication rate. The slope of the regression line (in green) is large (steep line), which shows that the predictor has a strong influence on the outcome variable [35,36,37,38,39,41,42,43,44,46,48,49,54,55,57,58,59,60,65,66,70,74,75].

Surgical approach: Of the 23 RCTs [35,36,37,38,39,41,42,43,44,46,48,49,54,55,57,58,59,60,65,66,70,74,75] with 2152 THAs reporting the surgical approach, a negative association (predictor estimate = −1.72) was found between SuperPATH and the complication rate. For each 1 percentage point increase in the frequency of SuperPATH surgical technique, the effect size for the complication rate decreased by an average of 1.72 (*p* < 0.01; Figure 13).

The remaining risk factors and predictors (patient age, BMI, preoperative HHS, sex, dysplasia, ANFH, other surgical approach, except SuperPATH, operation time, incision length, intraoperative blood loss, acetabular cup inclination, CRP 1–3 days postoperatively, CK 1–3 days postoperatively, and use of bone cement) were not statistically significant. All results of the meta-regression analysis for all risk factors and predictors and all outcome parameters are shown in Table 5.

## 4. Discussion

Our systematic review and meta-regression analysis examined risk factors and predictors for the effect size of the functional outcome and complication rate of minimally invasive and conventional approach THA, including 3630 THAs in 3607 patients from 41 RCTs. For a better understanding, it must be emphasized once again that the present study does not simply examine the factors influencing THA, but examines which factors influence the differences in the outcome between the minimally invasive and conventional approach THA. To the best of our knowledge, this is the first study of its kind. The main results of our study showed that minimally invasive THA had a better short-term functional outcome than conventional approach THA. There was no difference in short-term complications. We identified patient age, ANFH, and incision length as predictors of the effect size of the HHS ≥ 6 months postoperatively. We identified osteoarthritis, femoral neck fracture, and SuperPATH surgical technique as predictors of the effect size of the complication rate.

The outcome parameter HHS ≤ 3 months postoperatively showed a high risk of publication bias, while the outcome parameters HHS ≥ 6 months postoperatively and the complication rate showed a low risk of publication bias. A total of 19 out of 45 RCTs had a high risk of bias, 8 RCTs had a moderate risk of bias and 14 RCTs had a low risk of bias. The outcome parameters HHS ≤ 3 months postoperatively and complication rate showed low quality of evidence, the outcome parameter HHS ≥ 6 months postoperatively showed moderate quality of evidence. The meta-analysis of the outcome parameters did not show relevant differences between THA through minimally invasive approaches compared with THA through conventional approaches. Minimally invasive THA had 3.93 and 1.62 points higher HHS ≤ 3 months postoperatively and ≥ 6 months postoperatively, respectively, than the conventional approach THA. These small differences in hip function are clinically irrelevant. Minimally invasive THA was indifferent compared with the complication risk of conventional approach THA. We considered 15 potential risk factors and predictors. We did not identify any risk factors and predictors for the effect size of the outcome parameter HHS ≤ 3 months postoperatively. The effect size of the outcome parameter HHS ≥ 6 months postoperatively was influenced by the patient age, ANFH, and incision length. The effect size of the complication rate was influenced by osteoarthritis, femoral neck fracture, and SuperPATH surgical technique.

Patient age and HHS ≥ 6 months postoperatively showed a positive association. For each 1-year increase in mean age, the effect size for HHS ≥ 6 months postoperatively increased by an average of 0.14 points. This means that the difference found in the meta-analysis between minimally invasive and conventional approach THA changes by 0.14 HHS points per one patient-year in favour of the minimally invasive approaches. Elderly patients therefore benefit more from minimally invasive THA than from conventional approach THA compared with younger patients. This finding can be explained by the fact that minimally invasive approaches are muscle-sparing and less traumatic. In general, younger patients are better able to compensate for tissue trauma than elderly patients. The specialist literature is conflicting on this issue. A 2016 systematic review by Buirs et al. [25] found a significant negative association between patient age and functional outcome. Another systematic review by Hofstede et al. [26] found no relevant influence of patient age on THA outcome. A retrospective study of 1806 patients by Huddleston et al. [23] showed that increasing patient age is a risk factor for experiencing any adverse event. A matched case-control study by Melloh et al. [24] showed that elderly patients had a lower risk of cemented stem loosening, with the odds decreasing by 3.00% per year of age. A systematic review by Prokopetz et al. [27] showed a statistically significant association between patient age and revision risk. The younger patients had an increased risk of revision, while the risk generally decreased with each additional decade of age. A meta-analysis by Ren et al. [28] showed that age was not strongly associated with the infection risk.

The ANFH and HHS ≥ 6 months postoperatively showed a negative association. For every 1 percentage point increase in the incidence of ANFH, the effect size for HHS ≥ 6 months postoperatively decreased by an average of 0.03 points. This means that the difference found in the meta-analysis between minimally invasive and conventional approach THA levels off as the incidence of ANFH increases. The finding that the choice between minimally invasive or conventional approach does not play a relevant role in ANFH is important for surgical practice. This finding has not yet been described in the specialist literature. The systematic review by Prokopetz et al. [27] showed a higher revision risk in patients diagnosed with ANFH as compared with osteoarthritis.

Incision length and HHS ≥ 6 months postoperatively showed a negative association. For each 1 cm increase in mean incision length, the effect size for HHS ≥ 6 months postoperatively decreased by an average of 0.82 points. This means that the difference found in the meta-analysis between minimally invasive and conventional approach THA changes by 0.82 points for every 1 cm increase in incision length to the disadvantage of the minimally invasive approaches. In our patient cohort, the mean incision length of THA through conventional approaches was 12.86 cm, and the mean incision length of THA through minimally invasive approaches was 9.10 cm. In general, minimally invasive THA aims to achieve an incision length ≤ 10 cm, which explains why the difference in the HHS levels off as the incision length increases.

Osteoarthritis and the complication rate showed a positive association. For every 1 percentage point increase in the incidence of osteoarthritis, the effect size for the complication rate increased by an average of 0.02. Femoral neck fracture and the complication rate showed a negative association. For every 1 percentage point increase in the incidence of femoral neck fracture, the effect size for the complication rate decreased by an average of 0.02. These changes in the difference in the complication rate between minimally invasive and conventional approach THA do not appear to be clinically relevant. The meta-analysis by Ren et al. [28] found a higher infection risk in patients with femoral neck fractures.

SuperPATH surgical technique and the complication rate showed a negative association. For each 1 percentage point increase in the frequency of SuperPATH, the effect size for the complication rate decreased by an average of 1.72. The more often SuperPATH was used as a minimally invasive approach, the more significantly the complication rate of minimally invasive THA decreased compared with the conventional approach. A recently published meta-analysis by Ramadanov [80] showed no differences in complication rates between SuperPATH and conventional approaches in THA.

Based on these findings, we recommend that more frequent use of minimally invasive THA in elderly patients should be considered. However, there are still some potential disadvantages of the minimally invasive THA that should be taken into account when choosing the approach and technique for a particular patient. The minimally invasive approach aims to achieve a shorter incision length, which results in a limited view of the surgical field, making it more difficult to identify anatomical abnormalities or complications during surgery. There is an increased risk of nerve and vessel injury, which can be minimized by the technical skill of the surgeon. In addition, minimally invasive approaches have a longer operation time than conventional approaches due to the need to use specialized instruments and the complexity of the surgical technique itself. Longer operation times may increase the risk of bacterial contamination and infection. With regard to the latter, prosthesis infection can also result from the complex interaction of several factors: bacteria, prosthesis, and host weakness. Again, it depends on the surgical skill of the surgeon to keep the operation time as short as possible in minimally invasive THA, since excessive operation times of more than 180 min. after joint replacement are associated with significant blood loss (more than 800 mL), blood transfusion, excessive tissue trauma, the presence of nosocomial bacterial strains, and failure to follow the rules of asepsis and antisepsis [81].

We identified the following strengths and limitations of our meta-regression analysis: (1) We used an intention-to-treat (ITT) analysis, so a certain number of patients were lost to follow-up. (2) The medium-term and long-term THA outcomes were not considered. (3) Some risk factors and predictors were reported less frequently than others. Further meta-regression analyses with larger data sets are needed to draw definitive conclusions on these risk factors and predictors. (4) In some cases, information on standard deviation was not reported, and it was inserted via imputation. (5) We examined a wide range of risk factors and predictors and performed a meta-regression analysis, which has not been performed before on this topic.

## 5. Conclusions

We identified patient age, ANFH, and incision length as predictors of the effect size of the HHS ≥ 6 months postoperatively; and osteoarthritis, femoral neck fracture, and SuperPATH surgical technique as predictors for the effect size of the complication rate. Elderly patients seem to benefit from minimally invasive THA. SuperPATH seems to reduce the complication rate of minimally invasive THA compared with conventional approach THA. Based on these findings, and taking into account our limitations, we recommend that more frequent use of minimally invasive THA in elderly patients should be considered.

## Figures and Tables

**Table 1:** Main characteristics of the included RCTs and patients (See continuation in Table 2). RCT: randomized controlled trial; THA: total hip arthroplasty; SD: standard deviation; BMI: body mass index; HHS: Harris Hip Score; MI: minimally invasive; AL: anterolateral; DAA: direct anterior approach; L: lateral; MH: MicroHip; P: posterior; PL: posterolateral; S: SuperPATH; CA: conventional approach; TT: traction table; Lat: lateral decubitus position; NR: not reported; * This RCT divided the patient cohort according to their BMI; ** This RCT divided the patient cohort according to their diagnosis; *** Both RCTs used identical patient cohorts, giving different outcome parameters.

RCT	Year of Publication, Origin	Patients, N	THAs, N	Sex, Male, N (%)	Approach	Use of Bone Cement, N	Table/ Patient Position	Mean Age, Years, SD	Mean BMI, kg/m^2^, SD	HHS Preoperatively, Points, SD
Barrett WP et al. [35]	2013, USA	43	43	29 (67.44)	MI DAA	0	TT	61.40 ± 9.20	30.70 ± 5.40	57.60 ± 10.20
		44	44	19 (43.18)	CA PL	0	Lat	63.20 ± 7.70	29.10 ± 5.00	55.10 ± 9.10
Bon G et al. [36]	2019, France	50	50	21 (42.00)	MI DAA	7	TT	67.26 ± 10.00	26.46 ± 3.58	54.04 ± 14.94
		50	50	23 (46.00)	CA P	7	NR	68.98 ± 7.93	26.69 ± 3.12	52.31 ± 13.06
Brismar BH et al. [37]	2018, Sweden	50	50	18 (36.00)	MI DAA	0	Supine	66.00 ± 4.00	27.00 ± 1.25	NR
		50	50	17 (34.00)	CA L	0	Lat	67.00 ± 4.00	27.00 ± 1.50	NR
Cheng TE et al. [38]	2016, Australia	35	35	15 (42.29)	MI DAA	NR	TT	59.00 ± 3.75	27.70 ± 1.05	NR
		37	37	18 (48.65)	CA P	NR	Lat	62.50 ± 3.50	28.30 ± 1.57	NR
D’Arrigo C et al. [39]	2009, Italy	20	20	12 (60.00)	MI DAA	0	NR	64.00 ± 8.00	22.70 ± 1.50	37.70 ± 19.00
		149	149	81 (54.36)	CA L	0	NR	65.00 ± 9.80	28.00 ± 1.80	39.00 ± 10.20
De Anta-Diaz B et al. [40]	2016, Spain	49	49	26 (53.06)	MI DAA	8	NR	64.80 ± 10.10	26.60 ± 3.90	44.40 ± 13.60
		50	50	26 (52.00)	CA L	6	NR	63.50 ± 12.50	26.90 ± 3.10	42.90 ± 15.20
Dienstknecht T et al. [41] *	2013, Germany	42	42	14 (33.33)	MI MH	2	Lat	61.00 ± 13.00	26.10 ± 3.00	48.00 ± 15.00
		36	36	12 (33.33)	CA L	1	NR	62.00 ± 13.00	24.30 ± 3.60	46.00 ± 16.00
		41	41	24 (58.54)	MI MH	3	Lat	61.00 ± 11.00	34.30 ± 4.40	44.00 ± 15.00
		15	15	10 (66.67)	CA L	0	NR	61.00 ± 10.00	34.60 ± 4.10	46.00 ± 16.00
Fink B et al. [42]	2010, Germany	50	50	25 (50.00)	MI P	50	NR	71.90 ± 6.10	27.00 ± 4.80	NR
		50	50	23 (46.00)	CA PL	50	NR	71.50 ± 5.60	28.00 ± 3.80	NR
Gao P et Shi X [43]	2020, China	35	35	23 (65.71)	MI S	NR	Lat	69.26 ± 3.28	23.09 ± 2.57	15.43 ± 2.92
		35	35	20 (57.14)	CA P	NR	Lat	68.81 ± 3.45	23.21 ± 2.44	15.65 ± 2.71
Hou JZ et al. [44]	2017, China	20	20	13 (65.00)	MI S	NR	Lat	54.30 ± 13.70	24.50 ± 3.60	33.80 ± 5.40
		20	20	12 (60.00)	CA	NR	Lat	53.80 ± 12.90	23.90 ± 4.10	31.90 ± 6.10
Huang K et al. [45] **	2021, China	37	37	31 (83.78)	MI S	NR	Lat	56.20 ± 11.50	NR	47.30 ± 6.10
		58	58	50 (86.21)	CA L	NR	Lat	53.00 ± 10.40	NR	45.70 ± 8.10
		16	16	2 (12.50)	MI S	NR	Lat	78.10 ± 7.80	NR	40.60 ± 11.50
		18	18	8 (44.44)	CA L	NR	Lat	77.70 ± 10.10	NR	40.90 ± 11.60
Khan RJK et al. [46]	2012, Australia	44	44	24 (54.55)	MI P	44	Lat	72.30 ± 1.00	28.50 ± 0.70	NR
		45	45	19 (42.22)	CA P	45	Lat	72.80 ± 1.10	28.90 ± 0.60	NR
Li L [47]	2020, China	30	30	16 (53.33)	MI S	NR	Lat	70.35 ± 4.26	NR	25.41 ± 2.41
		30	30	18 (60.00)	CA PL	NR	Lat	70.12 ± 4.78	NR	26.35 ± 2.47
Ling Z et al. [48]	2020, China	50	50	31 (62.00)	MI S	NR	NR	89.14 ± 3.60	NR	46.08 ± 3.29
		50	50	29 (58.00)	CA PL	NR	NR	88.95 ± 3.71	NR	45.88 ± 3.71
Liu Y et al. [49]	2021, China	47	47	26 (55.32)	MI S	NR	Lat	68.27 ± 3.71	NR	67.70 ± 7.30
		47	47	24 (51.06)	CA PL	NR	Lat	68.55 ± 3.40	NR	68.66 ± 6.22
Liu W et al. [50]	2022, China	30	30	17 (56.67)	MI S	NR	Lat	58.59 ± 4.32	NR	58.73 ± 4.31
		30	30	18 (60.00)	CA	NR	Lat	58.31 ± 4.57	NR	58.79 ± 4.33
Martin R et al. [51]	2011, Belgium	42	42	12 (28.57)	MI AL	42	Lat	66.70 ± 10.10	30.60 ± 6.10	37.40 ± 15.50
		41	41	14 (34.15)	CA L	41	NR	63.10 ± 10.20	29.40 ± 5.50	40.20 ± 12.90
Meng W et al. [52]	2020, China	2	4	2 (100.00)	MI S	NR	Lat	51.00 ± 4.54	21.49 ± 1.73	37.86 ± 13.27
		2	4	2 (100.00)	CA PL	NR	Lat	51.00 ± 4.54	21.49 ± 1.73	37.66 ± 7.02
Mjaaland KE et al. [53] ***	2015, Norway	83	83	25 (30.12)	MI DAA	83	Supine	67.20 ± 8.60	27.70 ± 3.60	53.60 ± 13.70
		80	80	30 (37.50)	CA L	80	Lat	65.60 ± 8.60	27.60 ± 3.90	56.00 ± 11.20
Mjaaland KE et al. [54] ***	2018, Norway	83	83	25 (30.12)	MI DAA	83	Supine	67.20 ± 8.60	27.70 ± 3.60	53.60 ± 13.70
		80	80	30 (37.50)	CA L	80	Lat	65.60 ± 8.60	27.60 ± 3.90	56.00 ± 11.20
Moerenhout K et al. [55]	2019, Canada	28	28	11 (39.29)	MI DAA	0	TT	70.40 ± 9.10	27.60 ± 4.40	52.10 ± 19.70
		27	27	18 (66.67)	CA P	0	Lat	69.00 ± 8.80	26.50 ± 4.30	48.20 ± 10.10
Müller M et al. [56]	2010, Germany	21	21	12 (57.14)	MI AL	0	NR	66.00 ± 6.75	28.00 ± 4.25	55.90 ± 8.00
		16	16	8 (50.00)	CA L	0	NR	64.00 ± 13.75	26.00 ± 2.50	55.60 ± 12.00
Nistor DV et al. [57]	2017, Romania	35	35	26 (74.29)	MI DAA	0	Supine	67.00 ± 4.75	27.45 ± 3.76	NR
		35	35	16 (45.71)	CA L	0	Supine	64.00 ± 3.25	28.63 ± 3.12	NR
Ouyang C et al. [58]	2018, China	12	12	8 (66.67)	MI S	NR	Lat	54.00 ± 6.50	23.10 ± 2.30	45.67 ± 5.93
		12	12	9 (75.00)	CA PL	NR	Lat	55.00 ± 5.00	23.90 ± 3.38	46.92 ± 8.94
Pan F et al. [59]	2020, China	58	58	34 (58.62)	MI S	NR	Lat	65.23 ± 6.84	22.24 ± 4.15	83.85 ± 2.71
		58	58	33 (56.90)	CA PL	NR	Lat	65.62 ± 6.96	22.56 ± 4.22	84.02 ± 3.15
Reichert et al. [60]	2018, Germany	77	77	45 (58.44)	MI DAA	4	Supine	63.20 ± 8.20	28.10 ± 3.70	54.00 ± 14.20
		71	71	39 (54.93)	CA L	5	Supine	61.90 ± 7.80	28.30 ± 3.40	53.00 ± 15.70
Ren D et al. [61]	2016, China	21	21	12 (57.14)	MI S	NR	NR	57.96 ± 6.89	NR	35.35 ± 4.85
		21	21	13 (61.90)	CA	NR	NR	58.45 ± 6.25	NR	36.23 ± 3.54
Restreppo C et al. [62]	2010, USA	50	50	17 (34.00)	MI DAA	0	Supine	62.02 ± 12.38	25.18 ± 11.1	51.86 ± 7.88
		50	50	22 (44.00)	CA L	0	Supine	59.91 ± 9.00	25.17 ± 2.48	54.95 ± 5.53
Rykov K et al. [63]	2017, Netherlands	23	23	8 (34.78)	MI DAA	23	Supine	62.80 ± 6.10	29.00 ± 5.60	52.00 ± 6.67
		23	23	11 (47.83)	CA PL	23	Lat	60.20 ± 8.10	29.30 ± 4.80	51.00 ± 8.95
Schwarze M et al. [64]	2017, Germany	22	22	13 (59.09)	MI AL	0	Supine	59.00 ± 9.00	26.70 ± 4.20	53.00 ± 12.00
		21	21	13 (61.90)	CA L	0	Supine	59.00 ± 9.00	26.70 ± 4.20	59.00 ± 15.00
Taunton M et al. [65]	2014, USA	27	NR	12 (44.44)	MI DAA	NR	Supine	62.05 ± NR	27.70 ± NR	55.00 ± 4.25
		27	NR	13 (48.15)	CA P	NR	Lat	66.40 ± NR	29.20 ± NR	51.00 ± 6.00
Taunton M et al. [66]	2018, USA	52	52	27 (51.92)	MI DAA	0	NR	65.00 ± 10.00	29.00 ± 2.20	57.00 ± 13.00
		49	49	25 (51.02)	CA P	0	NR	64.00 ± 11.00	30.00 ± 4.00	56.00 ± 12.00
Varela-Egocheaga JR et al. [67]	2013, Spain	25	25	12 (48.00)	MI L	0	NR	64.80 ± 10.45	28.27 ± 3.67	52.70 ± 12.90
		25	25	12 (48.00)	CA L	0	NR	63.80 ± 9.70	27.78 ± 3.24	51.30 ± 14.90
Wang Z et Ge W [68]	2021, China	43	43	26 (60.47)	MI S	NR	Supine	71.53 ± 3.76	22.47 ± 1.12	62.18 ± 5.23
		42	42	24 (57.14)	CA PL	NR	Lat	71.58 ± 3.79	22.51 ± 1.15	62.65 ± 6.59
Xiao C et al. [69]	2021, China	49	49	16 (32.65)	MI P	0	Lat	71.06 ± 10.87	26.73 ± 4.18	NR
		57	57	26 (45.61)	CA PL	0	Lat	73.93 ± 10.02	26.39 ± 4.64	NR
Xie J et al. [70]	2017, China	46	46	12 (26.09)	MI S	0	Lat	66.60 ± 11.88	23.62 ± 1.63	28.90 ± 11.32
		46	46	19 (41.30)	CA P	0	Lat	64.47 ± 12.09	24.06 ± 2.72	29.30 ± 17.40
Yan T et al. [71]	2017, China	64	70	29 (45.21)	MI S	NR	NR	66.00 ± 4.00	24.50 ± 3.45	33.50 ± 5.30
		90	103	42 (46.67)	CA L	NR	NR	65.00 ± 6.50	23.60 ± 3.58	30.70 ± 7.60
Yang C et al. [72]	2010, China	55	55	26 (47.27)	MI AL	0	Lat	59.47 ± 13.24	23.12 ± 3.23	25.93 ± 11.30
		55	55	30 (54.55)	CA PL	0	Lat	55.82 ± 13.91	22.42 ± 3.95	28.18 ± 13.73
Yuan H et al. [73]	2018, China	40	40	24 (60.00)	MI S	0	Lat	74.30 ± 3.00	22.73 ± 1.71	33.00 ± 1.89
		44	44	21 (47.72)	CA PL	0	Lat	75.70 ± 3.25	22.36 ± 2.72	32.70 ± 1.32
Zhang ZL et al. [74]	2019, China	27	27	10 (37.04)	MI S	NR	NR	62.41 ± 6.44	24.53 ± 5.31	35.60 ± 8.80
		27	27	12 (44.44)	CA PL	NR	NR	61.28 ± 6.70	23.93 ± 4.89	36.20 ± 9.20
Zhao HY et al. [75]	2017, China	60	60	24 (40.00)	MI DAA	NR	Supine	64.88 ± 12.13	24.35 ± 3.10	40.19 ± 9.23
		60	60	22 (36.67)	CA PL	NR	Lat	62.18 ± 14.72	25.58 ± 2.83	43.11 ± 15.59

**Table 2:** Main characteristics of the patients (Continuation of Table 1). RCT: randomized controlled trial; ANFH: avascular necrosis of the femoral head; SD: standard deviation; CRP: c-reactive protein; CK: creatine kinase; NR: not reported * This RCT divided the patient cohort according to their BMI; ** This RCT divided the patient cohort according to their diagnosis; *** Both RCTs used identical patient cohorts, giving different outcome parameters.

RCT	Osteoarthritis, N	Femoral Neck Fracture, N	Dysplasia, N	ANFH, N	Operation Time, min., SD	Incision Length, cm, SD	Intraoperative Blood Loss, mL, SD	Acetabular Cup Inclination Angle, Degree, SD	CRP 1–3 Days Postoperatively, mg/L, SD	CK 1–3 Days Postoperatively, U/L, SD
Barrett WP et al. [35]	43	0	0	0	84.30 ± 12.40	13.70 ± 0.90	391.00 ± 206.00	47.10 ± 6.10	NR	NR
	44	0	0	0	60.50 ± 12.40	12.70 ± 1.30	191.00 ± 107.00	42.40 ± 7.60	NR	NR
Bon G et al. [36]	50	0	0	0	70.10 ± 11.00	NR	NR	37.74 ± 4.20	NR	NR
	50	0	0	0	56.70 ± 11.79	NR	NR	39.60 ± 6.87	NR	NR
Brismar BH et al. [37]	50	0	0	0	101.00 ± NR	NR	325.00 ± NR	NR	NR	NR
	50	0	0	0	80.00 ± NR	NR	300.00 ± NR	NR	NR	NR
Cheng TE et al. [38]	35	0	0	0	125.00 ± NR	10.70 ± 8.00	NR	46.20 ± NR	NR	NR
	37	0	0	0	100.00 ± NR	13.50 ± 7.00	NR	45.90 ± NR	NR	NR
D’Arrigo C et al. [39]	20	0	0	0	121.00 ± 23.60	NR	1344.00 ± 710.00	NR	NR	NR
	149	0	0	0	77.00 ± 15.10	NR	1644.00 ± 757.70	NR	NR	NR
De Anta-Diaz B et al. [40]	49	0	0	0	78.20 ± 16.20	10.40 ± 0.90	NR	NR	11.40 ± 5.20	203.20 ± 53.70
	50	0	0	0	82.20 ± 15.20	11.50 ± 0.70	NR	NR	14.40 ± 9.10	387.00 ± 174.00
Dienstknecht T et al. [41] *	42	0	0	0	66.00 ± 27.00	13.00 ± 2.00	440.00 ± 821.00	49.20 ± 7.00	142.00 ± 56.00	NR
	36	0	0	0	58.00 ± 15.00	9.00 ± 1.00	346.00 ± 170.00	48.20 ± 6.10	118.00 ± 53.00	NR
	41	0	0	0	70.00 ± 28.00	14.00 ± 3.00	383.00 ± 265.00	50.10 ± 5.00	149.00 ± 62.00	NR
	15	0	0	0	60.00 ± 9.00	9.00 ± 1.00	302.00 ± 138.00	48.10 ± 6.00	178.00 ± 115.00	NR
Fink B et al. [42]	44	0	1	5	51.90 ± 11.40	NR	262.70 ± 149.70	43.70 ± 5.90	NR	NR
	44	0	1	5	50.90 ± 10.20	NR	382.00 ± 179.90	42.80 ± 6.60	NR	NR
Gao P et Shi X [43]	0	35	0	0	68.59 ± 5.37	7.41 ± 0.85	88.68 ± 6.04	NR	NR	NR
	0	35	0	0	61.56 ± 6.02	14.55 ± 1.86	208.52 ± 4.61	NR	NR	NR
Hou JZ et al. [44]	6	0	0	14	115.00 ± 10.09	7.20 ± 0.50	315.00 ± 116.00	43.90 ± 2.90	NR	NR
	5	0	0	15	105.00 ± 15.40	15.00 ± 1.60	470.00 ± 127.10	44.70 ± 3.10	NR	NR
Huang K et al. [45] **	0	0	0	37	82.80 ± 14.30	7.62 ± 1.11	71.90 ± 17.90	NR	NR	NR
	0	0	0	58	73.50 ± 23.20	10.64 ± 1.16	174.70 ± 50.50	NR	NR	NR
	0	16	0	0	83.70 ± 27.00	7.63 ± 1.20	72.50 ± 16.90	NR	NR	NR
	0	18	0	0	75.10 ± 19.80	10.33 ± 1.08	162.80 ± 48.50	NR	NR	NR
Khan RJK et al. [46]	42	0	0	2	87.00 ± 2.97	12.60 ± 0.72	NR	41.80 ± 1.02	98.20 ± 53.50	NR
	43	0	0	2	90.00 ± 2.12	19.30 ± 0.37	NR	45.30 ± 0.98	92.00 ± 47.25	NR
Li L [47]	NR	NR	NR	NR	NR	NR	NR	NR	NR	NR
	NR	NR	NR	NR	NR	NR	NR	NR	NR	NR
Ling Z et al. [48]	0	50	0	0	118.25 ± 16.95	7.06 ± 0.99	185.47 ± 20.23	NR	NR	NR
	0	50	0	0	68.81 ± 10.37	9.13 ± 1.18	388.95 ± 47.71	NR	NR	NR
Liu Y et al. [49]	0	47	0	0	NR	7.32 ± 1.30	92.43 ± 7.14	NR	NR	NR
	0	47	0	0	NR	13.30 ± 2.46	195.83 ± 18.99	NR	NR	NR
Liu W et al. [50]	3	13	0	14	88.83 ± 7.36	7.83 ± 0.36	203.03 ± 23.14	NR	NR	NR
	6	9	0	15	90.29 ± 7.27	12.29 ± 1.27	387.49 ± 24.25	NR	NR	NR
Martin R et al. [51]	37	0	0	5	114.12 ± 21.47	9.50 ± 1.40	NR	NR	14.20 ± 7.40	NR
	37	0	0	4	95.78 ± 18.53	14.80 ± 3.30	NR	NR	13.80 ± 5.70	NR
Meng W et al. [52]	0	0	0	4	103.25 ± 12.41	7.62 ± 0.97	1108.50 ± 163.63	38.75 ± 8.21	NR	NR
	0	0	0	4	66.50 ± 13.79	11.12 ± 1.21	843.50 ± 111.60	44.50 ± 3.64	NR	NR
Mjaaland KE et al. [53] ***	83	0	0	0	77.00 ± 21.00	9.50 ± 1.25	NR	NR	47.50 ± 39.30	989.50 ± 446.70
	80	0	0	0	62.00 ± 10.75	13.50 ± 1.25	NR	NR	50.00 ± 41.50	965.80 ± 467.80
Mjaaland KE et al. [54] ***	83	0	0	0	NR	NR	NR	NR	NR	NR
	80	0	0	0	NR	NR	NR	NR	NR	NR
Moerenhout K et al. [55]	NR	0	0	NR	59.90 ± 12.70	NR	NR	43.30 ± 8.40	NR	NR
	NR	0	0	NR	45.70 ± 17.90	NR	NR	39.80 ± 5.40	NR	NR
Müller M et al. [56]	21	0	0	0	51.00 ± 6.80	8.00 ± 1.60	NR	NR	NR	NR
	16	0	0	0	50.00 ± 7.40	10.40 ± 2.00	NR	NR	NR	NR
Nistor DV et al. [57]	35	0	0	0	70.00 ± 1.25	12.18 ± 1.91	NR	36.97 ± 1.85	NR	469.00 ± 83.00
	35	0	0	0	70.00 ± 3.75	14.79 ± 2.25	NR	39.63 ± 2.88	NR	357.00 ± 56.25
Ouyang C et al. [58]	5	0	0	7	109.60 ± 28.30	10.40 ± 3.00	138.33 ± 42.82	37.08 ± 6.53	63.27 ± 49.43	661.75 ± 261.01
	6	0	0	6	67.50 ± 16.20	12.50 ± 1.40	141.67 ± 35.89	39.67 ± 6.95	87.55 ± 38.94	1035.25 ± 540.62
Pan F et al. [59]	12	26	NR	15	92.58 ± 12.35	7.51 ± 0.82	NR	NR	NR	NR
	11	25	NR	18	125.32 ± 12.63	15.23 ± 2.14	NR	NR	NR	NR
Reichert et al. [60]	77	0	0	0	NR	NR	NR	38.60 ± 5.70	NR	NR
	71	0	0	0	NR	NR	NR	40.28 ± 6.20	NR	NR
Ren D et al. [61]	0	0	0	21	NR	NR	NR	NR	NR	NR
	0	0	0	21	NR	NR	NR	NR	NR	NR
Restreppo C et al. [62]	50	0	0	0	56.42 ± 13.75	NR	172.50 ± 137.50	NR	NR	NR
	50	0	0	0	54.88 ± 16.00	NR	170.00 ± 112.50	NR	NR	NR
Rykov K et al. [63]	23	0	0	0	71.00 ± 7.00	NR	325.70 ± 99.74	NR	NR	NR
	23	0	0	0	62.00 ± 7.00	NR	273.70 ± 181.00	NR	NR	NR
Schwarze M et al. [64]	22	0	0	0	70.00 ± 20.00	NR	NR	NR	NR	NR
	21	0	0	0	67.00 ± 18.00	NR	NR	NR	NR	NR
Taunton M et al. [65]	27	0	0	0	NR	NR	NR	38.00 ± 1.25	NR	NR
	27	0	0	0	NR	NR	NR	40.00 ± 1.50	NR	NR
Taunton M et al. [66]	52	0	0	0	NR	NR	NR	37.00 ± 5.00	NR	NR
	49	0	0	0	NR	NR	NR	39.00 ± 6.00	NR	NR
Varela-Egocheaga JR et al. [67]	21	0	0	4	62.04 ± NR	NR	NR	43.70 ± NR	NR	NR
	22	0	0	3	60.60 ± NR	NR	NR	45.30 ± NR	NR	NR
Wang Z et Ge W [68]	0	43	0	0	105.79 ± 18.75	8.26 ± 1.02	89.47 ± 9.32	NR	NR	NR
	0	42	0	0	73.16 ± 9.82	11.19 ± 0.93	253.86 ± 42.58	NR	NR	NR
Xiao C et al. [69]	0	49	0	0	84.47 ± 19.37	9.10 ± 0.94	NR	NR	97.21 ± 39.27	370.23 ± 249.37
	0	57	0	0	105.44 ± 10.50	15.56 ± 1.20	NR	NR	113.29 ± 4.98	504.62 ± 21.88
Xie J et al. [70]	46	0	0	0	103.60 ± 11.80	7.40 ± 1.06	303.60 ± 106.30	43.60 ± 6.80	NR	NR
	46	0	0	0	106.50 ± 16.50	14.50 ± 2.38	326.40 ± 127.20	44.50 ± 6.50	NR	NR
Yan T et al. [71]	14	11	0	39	52.00 ± 5.00	5.80 ± 0.60	349.00 ± 28.00	38.90 ± 2.60	NR	NR
	12	23	0	55	36.00 ± 15.00	14.30 ± 1.20	165.00 ± 70.00	39.50 ± 0.40	NR	NR
Yang C et al. [72]	12	11	0	32	77.55 ± 13.39	7.49 ± 0.86	376.18 ± 168.30	48.30 ± 5.30	NR	NR
	19	13	0	23	73.67 ± 14.51	15.19 ± 1.82	605.00 ± 225.12	48.90 ± 6.60	NR	NR
Yuan H et al. [73]	5	21	4	10	57.50 ± 5.66	7.50 ± 1.13	175.00 ± 11.32	NR	NR	NR
	6	24	2	12	63.64 ± 6.50	10.73 ± 1.30	209.09 ± 16.96	NR	NR	NR
Zhang ZL et al. [74]	7	0	5	15	NR	NR	NR	NR	NR	NR
	9	0	4	14	NR	NR	NR	NR	NR	NR
Zhao HY et al. [75]	41	0	6	13	83.26 ± 6.69	9.09 ± 0.45	165.89 ± 42.60	40.30 ± 2.80	NR	NR
	40	0	7	13	65.48 ± 13.32	13.14 ± 0.31	123.84 ± 56.83	41.80 ± 3.40	NR	NR

**Table 3:** Risk of bias assessment. (+): low risk of bias; (?): moderate risk of bias; (−): high risk of bias.

RCT	Bias Arising from the Randomization Process	Bias Due to Deviation from Intended Interventions	Bias Due to Missing Outcome Data	Bias in Measurement of the Outcome	Bias in Selection of the Reported Result	Overall Risk of Bias
Barrett WP et al. [35]	+	−	?	?	+	−
Bon G et al. [36]	+	+	+	+	+	+
Brismar BH et al. [37]	+	+	−	+	+	−
Cheng TE et al. [38]	+	+	−	+	+	−
D’Arrigo C et al. [39]	+	+	+	+	+	+
De Anta-Diaz B et al. [40]	−	+	+	+	+	−
Dienstknecht T et al. [41]	−	+	+	+	+	−
Fink B et al. [42]	+	+	+	+	+	+
Gao P and Shi X [43]	+	?	−	+	+	−
Hou JZ et al. [44]	+	?	+	+	+	?
Huang K et al. [45]	−	?	+	+	+	−
Khan RJK et al. [46]	+	+	+	+	+	+
Li L [47]	+	?	−	−	+	−
Ling Z et al. [48]	?	+	−	+	+	−
Liu Y et al. [49]	+	+	−	+	+	−
Liu W et al. [50]	+	+	+	+	+	+
Martin R et al. [51]	?	?	+	+	?	?
Meng W et al. [52]	+	+	+	+	+	+
Mjaaland KE et al. [53]	+	+	+	+	+	+
Mjaaland KE et al. [54]	+	+	+	+	+	+
Moerenhout K et al. [55]	+	+	+	+	+	+
Müller M et al. [56]	+	+	?	?	+	?
Nistor DV et al. [57]	−	+	+	+	+	−
Ouyang C et al. [58]	+	+	+	+	+	+
Pan F et al. 2020 [59]	+	?	−	+	+	−
Reichert JC et al. [60]	−	+	+	+	+	−
Ren D et al. [61]	+	?	−	?	?	−
Restreppo C et al. [62]	+	+	+	+	+	+
Rykov K et al. [63]	+	+	−	+	+	−
Schwarze M et al. [64]	?	?	−	−	+	−
Taunton M et al. [65]	+	+	?	+	+	?
Taunton M et al. [66]	+	+	?	+	+	?
Varela-Egocheaga JR et al. [67]	−	−	+	+	+	−
Wang Z and Ge W [68]	+	?	−	+	+	−
Xiao C et al. [69]	?	+	+	+	+	?
Xie J et al. [70]	+	+	+	+	+	+
Yan T et al. [71]	+	?	?	+	+	?
Yang C et al. [72]	+	+	+	+	+	+
Yuan H et al. [73]	+	?	−	+	+	−
Zhang ZL et al. [74]	+	+	?	+	+	?
Zhao HY et al. [75]	+	+	+	+	+	+

**Table 4:** Level of evidence assessment according to GRADE recommendations. RCT: randomized controlled trial; HHS: Harris Hip Score; SD: standard deviation.

Number of RCTs	Design	Risk of Bias	Inconsistency	Indirectness	Imprecision	Other Considerations	Quality of Evidence
HHS ≤ 3 months postoperatively							
32	RCT	Serious	Serious	No serious indirectness	Serious	In some cases, SD was calculated via imputation	Low
HHS ≥ 6 months postoperatively							
21	RCT	Serious	No serious inconsistency	No serious indirectness	No serious imprecision	-	Moderate
Complication rate							
23	RCT	Serious	Serious	No serious indirectness	No serious imprecision	-	Low

**Table 5:** All results of the meta-regression analysis for all risk factors and predictors and all outcome parameters. *: statistically significant; **: The calculation is N/A because with a number of < 3 RCTs included, no meta-regression can be performed; RCT: randomized controlled trial; THA: total hip arthroplasty; HHS: Harris hip score; BMI: body mass index; ANFH: avascular necrosis of the femoral neck; CRP: C-reactive protein; CK: creatine kinase.

Outcome	Predictor	Number of RCTs	Number of THAs	I2	Tau2	QE.p.val	Predictor.Estimate	Predictor.p.val
**HHS ≤ 3 months postoperatively**	**Patient age**	32	2690	97.16	19.69	<0.01	0.06	0.71
	**BMI**	27	2305	95.47	17.40	<0.01	−0.22	0.54
	**Preoperative HHS**	31	2584	95.14	19.56	<0.01	−0.06	0.33
	**Sex**	32	2690	96.54	18.51	<0.01	0.13	0.16
	**Osteoarthritis**	30	2575	97.54	20.72	<0.01	−0.02	0.46
	**Femoral neck fracture**	31	2630	96.61	18.97	<0.01	0.03	0.16
	**Dysplasia**	12	820	79.58	4.12	<0.01	0.05	0.75
	**ANFH**	30	2575	97.24	20.91	<0.01	−0.02	0.59
	**Surgical approach**	32	2690	96.09	19.02	<0.01	1.86	0.27
	**Operation time**	25	2137	97.43	23.12	<0.01	0.05	0.40
	**Incision length**	19	1668	98.20	31.16	<0.01	−0.34	0.79
	**Intraoperative blood loss**	18	1625	97.70	29.86	<0.01	−0.01	0.31
	**Cup inclination**	15	1296	91.17	25.12	<0.01	0.27	0.52
	**CRP 1–3 days postoperatively**	4	363	30.24	1.22	0.53	−0.02	0.35
	**CK 1–3 days postoperatively**	3	229	8.63	0.17	0.53	0.01	0.49
	**Use of bone cement**	17	1561	94.47	7.59	<0.01	−0.01	0.74
**HHS ≥ 6 months postoperatively**	**Patient age**	21	1698	70.57	2.31	<0.01	0.14	0.01 *
	**BMI**	19	1538	60.08	1.42	0.57	0.19	0.12
	**Preoperative HHS**	21	1698	75.37	3.20	<0.01	0.01	0.91
	**Sex**	21	1698	73.45	2.95	<0.01	0.07	0.18
	**Osteoarthritis**	19	1583	71.71	2.57	0.01	0.01	0.69
	**Femoral neck fracture**	20	1638	68.33	2.22	0.01	0.02	0.13
	**Dysplasia**	12	814	71.47	3.12	0.01	−0.10	0.40
	**ANFH**	19	1583	68.11	2.02	0.01	−0.03	0.04 *
	**Surgical approach**	21	1698	77.47	3.21	<0.01	−0.09	0.93
	**Operation time**	16	1281	76.57	3.29	0.01	−0.01	0.75
	**Incision length**	13	1133	70.34	1.86	0.03	−0.82	0.03 *
	**Intraoperative blood loss**	9	798	83.27	3.08	0.01	−0.01	0.63
	**Cup inclination**	12	1022	32.72	0.63	0.79	−0.08	0.39
	**CRP 1–3 days postoperatively**	3	206	7.65	0.54	0.51	−0.03	0.44
	**CK 1–3 days postoperatively**	2	123	N/A **	N/A **	N/A **	N/A **	N/A **
	**Use of bone cement**	12	989	69.44	2.22	0.12	−0.02	0.48
**Complication rate**	**Patient age**	23	2152	62.37	1.90	0.01	−0.10	0.06
	**BMI**	21	1958	63.03	2.02	0.01	0.12	0.50
	**Preoperative HHS**	18	1721	52.28	1.27	0.02	0.01	0.74
	**Sex**	23	2152	65.41	2.14	<0.01	−0.04	0.32
	**Osteoarthritis**	22	2097	61.38	1.79	0.01	0.02	0.02 *
	**Femoral neck fracture**	23	2152	59.92	1.71	0.01	−0.02	0.02 *
	**Dysplasia**	11	859	32.41	0.60	0.38	−0.01	0.97
	**ANFH**	22	2097	68.06	2.34	<0.01	−0.01	0.98
	**Surgical approach**	23	2152	60.78	1.76	0.01	−1.72	0.02 *
	**Operation time**	17	1538	68.71	2.46	<0.01	0.02	0.31
	**Incision length**	13	1108	73.49	2.95	<0.01	0.27	0.39
	**Intraoperative blood loss**	12	1130	43.77	1.02	0.30	0.01	0.08
	**Cup inclination**	15	1286	63.82	2.35	0.01	0.01	0.94
	**CRP 1–3 days postoperatively**	3	247	12.64	0.21	0.48	−0.01	0.55
	**CK 1–3 days postoperatively**	2	94	N/A **	N/A **	N/A **	N/A **	N/A **
	**Use of bone cement**	13	1408	49.33	1.15	0.10	0.01	0.82

## Data Availability

Raw data set is available in the supplement.

## Supplementary Materials

The following supporting information can be downloaded at: https://www.mdpi.com/article/10.3390/jcm12185895/s1, Raw data set.

## Abbreviations

ANFH	avascular necrosis of the femoral head
BMI	body mass index
CI	confidence interval
CINAHL	Cumulative Index to Nursing and Allied Health Literature
CK	creatine kinase
CNKI	China National Knowledge Infrastructure
CRP	C-reactive protein
HHS	Harris Hip Score
ITT	intention-to-treat
MD	mean difference
OR	odds ratio
THA	total hip arthroplasty
